# The Low Proportion and Associated Factors of Involuntary Admission in the Psychiatric Emergency Service in Taiwan

**DOI:** 10.1371/journal.pone.0129204

**Published:** 2015-06-05

**Authors:** Jen-Pang Wang, Chih-Chiang Chiu, Tsu-Hui Yang, Tzong-Hsien Liu, Chia-Yi Wu, Pesus Chou

**Affiliations:** 1 Institute of Public Health and Community Medicine Research Center, National Yang-Ming University, Taipei, Taiwan; 2 Department of Psychiatry, Taipei City Psychiatric Center, Taipei City Hospital, Taipei, Taiwan; 3 Department of Healthcare Management, National Taipei University of Nursing and Health Sciences, Taipei, Taiwan; 4 Department of Psychiatry, School of Medicine, Taipei Medical University, Taipei, Taiwan; 5 Medical Affairs Division, Department of Health, Taipei City Government, Taipei, Taiwan; 6 Department of Nursing, College of Medicine, National Taiwan University, Taipei, Taiwan; Yokohama City University, JAPAN

## Abstract

**Background:**

The involuntary admission regulated under the Mental Health Act has become an increasingly important issue in the developed countries in recent years. Most studies about the distribution and associated factors of involuntary admission were carried out in the western countries; however, the results may vary in different areas with different legal and socio-cultural backgrounds.

**Aims:**

The aim of this study was to investigate the proportion and associated factors of involuntary admission in a psychiatric emergency service in Taiwan.

**Methods:**

The study cohort included patients admitted from a psychiatric emergency service over a two-year period. Demographic, psychiatric emergency service utilization, and clinical variables were compared between those who were voluntarily and involuntarily admitted to explore the associated factors of involuntary admission.

**Results:**

Among 2,777 admitted patients, 110 (4.0%) were involuntarily admitted. Police referrals and presenting problems as violence assessed by psychiatric nurses were found to be associated with involuntary admission. These patients were more likely to be involuntarily admitted during the night shift and stayed longer in the psychiatric emergency service.

**Conclusions:**

The proportion of involuntary admissions in Taiwan was in the lower range when compared to Western countries. Dangerous conditions evaluated by the psychiatric nurses and police rather than diagnosis made by the psychiatrists were related factors of involuntary admission. As it spent more time to admit involuntary patients, it was suggested that multidisciplinary professionals should be included in and educated for during the process of involuntary admission.

## Introduction

Although the provisions of psychiatric beds and psychiatric inpatient treatments have decreased in the European Union (EU) Member States since 1990, involuntary admission percentages or rates of psychiatric patients have increased over the same period [1]. These studies were mostly carried out nationally in the Netherlands [2] and England [3–6]. In 2000, the proportion of involuntary to total psychiatric hospitalizations, termed the involuntary admission quota, ranged from 3.2% to 26.4%, whereas the involuntary admission rate ranged from 6 to 218 with a median of 74 per 100,000 inhabitants across the EU [1, 7, 8]. However, only one study has been reported from the Asian country in South Korea where a high involuntary admission quota of 92% was noted in 2007 [9]. There remains a lack of studies about involuntary admissions in non-Western developed countries.

The patterns of involuntary placements to psychiatric hospitals vary considerably between countries or regions because of differences and changes in legal frameworks or procedures [7, 8, 10], provisions of psychiatric services [3, 6], patient characteristics [10], professional ethics and attitudes [8], societal pressures on psychiatrists away from libertarianism and toward coercion [4], and public’s preoccupation with the risk arising from mental illness [8]. In Taiwan, the involuntary admission rate was 7.3 per 100,000 residents from January 2009 to December 2010 according to involuntary admission data from the Department of Health and national population data from the Executive Yuan. This rate was in the lower range when compared to in other developed countries. According to the regulations of Mental Health Act 2007 in Taiwan, psychotic state, unable to comply with treatment, and danger-criterion are the legally defined set of required conditions for involuntary admission. The psychiatrists play roles in the initiation of emergency placement and application of involuntary admission within two days, while the regional commitment committees under the Department of Health which enroll multidisciplinary professionals are responsible for the approval of involuntary admission within three days [11]. These detained criteria and process of involuntary admission in Taiwan are different from the ones in other countries where evaluations are made by multidisciplinary professionals and broader criteria are required to apply for the involuntary admission [7, 8].

Besides the differences in the epidemiology of involuntary admission among mentally ill people across countries, different characteristics related to involuntary admission have been reported in previous studies. These can be categorized into socio-demographic, clinical, psychiatric service utilization, social support, and ecological factors. Relevant studies have been reviewed by the EU [1, 7, 9], and carried out in Asian countries [9, 12] and the USA [13]. Overall, these patients were characterized as younger or male gender, with more severe psychopathology, dangerous behaviors, poor insight or low motivation for treatment, and poor functioning or poor supportive system.

Therefore, the aim of this study was to investigate the proportion and associated factors of involuntary admission in the psychiatric emergency service in Taiwan. We hypothesized that not only the clinical characteristics assessed by the psychiatrists, but also clinical profiles evaluated by the psychiatric nurses, referral decisions made by the police, and psychiatric emergency service utilization characteristics are associated with coercive process of admission.

## Methods

### Study setting

The study cohort recruited patients visiting the psychiatric emergency service (PES) in the Taipei City Psychiatric Center (TCPC), which provides the majority of psychiatric emergency services in Taipei. This center provides comprehensive psychiatric services for the seven million residents in Taipei City and its satellite cities. One of the services is the psychiatric emergency services consisting of around 4,000 patient-visits per year. The PES has 16 observation beds, provides 24-hour services daily, and is staffed with on-site resident psychiatrists and psychiatric nurses, and on-call attending psychiatrists and social workers. The PES evaluates and manages patients from the community where around 40% of patients are involuntarily referred by the police, and 60% by their families or from other hospitals. The aims of this PES treatment are acute stabilization of psychiatric patients followed by further arrangements of appropriate psychiatric services. These include inpatient treatments which amount to around 40% of the total visits. From January 2009 to December 2010, 123 patients were compulsively admitted to TCPC, accounting for 43.5% of all 283 involuntary hospitalizations in Taipei City. Among the 123 involuntarily admitted patients, 110 were evaluated in the PES, while the others were assessed in the outpatient clinics or inpatient wards. The percentage of involuntary admissions compared to all inpatient episodes in TCPC during these two years was 2.4%, which was far less than in other countries.

### Study design and subjects

The study was approved by the Institutional Review Board of Taipei City Hospital (TCHIRB-101107). Study subjects were recruited from the clinical database of admitted patients evaluated in the PES of TCPC from January 2009 to December 2010. Exemption from informed consent was approved since the data were analyzed anonymously and reported. This database was originally established and recorded as part of clinical standard care by psychiatrists and psychiatric nurses. The collected data were assumed to be reliable and valid as every nurse and psychiatrist should mandatorily receive training courses of major topics related to emergency psychiatry every year. These included assessment of psychiatric triage, evaluation of presenting problems, and common diagnoses in the PES. These variables were reviewed and discussed by the whole team chaired by two attending psychiatrists in every morning meeting. It was then keyed in by the assistants of the Taipei psychiatric service network and reviewed by the attending psychiatrists and head nurse of the PES weekly.

### Study variables

Socio-demographic, clinical, and emergency service utilization variables were collected for all the study samples. Age and gender were included in the socio-demographic characteristics. The clinical characteristics included the triage level and presenting problems assessed by the psychiatric nurses when the patients visited the PES. The triage level was modified from the Canadian Triage and Acuity Scale (CTAS) [14] and categorized into four degrees of danger. Level IV was defined as imminent danger, level III as urgent danger, level II as less urgent, and level I as not urgent. Each risk level had its key assessment information, clinical examples, nursing actions, and timescales. Five presenting problems assessed by psychiatric nurses were chosen due to their relatedness with involuntary hospitalizations according to a literature review. These included violence, suicide, disturbance, substance use, and anxiety, which were operationally defined and assessed [15–19]. Violence and suicide were defined as either attempts or ideations of harming others or him/herself. Disturbance referred to behaviors which would bother others in various ways. Substance use would be applied when patients were suspected to be users of substance or alcohol before their arrivals. Patients who manifested anxious symptoms were recorded as anxiety. The diagnosis was made by the resident psychiatrists who were supervised by the attending psychiatrists according to the International Classification of Diseases, 9^th^ Revision, Clinical Modification (ICD-9-CM), which was the clinical assessment instrument as it had been the mandatory registry item of national health insurance claims in Taiwan. If any medical problem was considered, consultations with internists or neurologists were arranged. If the psychiatric conditions were evaluated as severe enough to require intensive management, the patients were transferred to the psychiatric intensive care unit (PICU). Regarding to the emergency service utilization patterns, referral of these patients in crises could be involuntarily made by the police and firefighters whose roles were regulated under the Mental Health Act [11] and the Police Power Exercise Act [20], while others voluntarily came to the PES individually or were accompanied by their families. Arrival time and discharge time were recorded and transformed into variables representing whether they arrived at or were discharged from the PES during the night shift (4 pm to 8 am). Length of PES stay was calculated in hours by subtracting the discharge time and arrival time. Referral location from catchment or non-catchment areas was also included in the emergency service utilization characteristics.

### Statistical analysis

Demographic, emergency service utilization, and clinical characteristics were described in both the total admitted and involuntarily admitted patients. Categorical variables were described in numbers with proportions for the total admitted patients and in numbers with rates in those involuntarily admitted. Continuous variables were presented in means with standard deviations in both the total and involuntarily admitted patients. Chi-square test was used to analyze whether there was statistical difference in involuntary admission rate among each categorical variable, while independent-samples t test was used to test the difference of mean in each continuous variable between those who were and not involuntarily admitted. Variables which showed a statistical difference in univariate analysis and those cited as a major related factor of involuntary admission in the past studies were entered into multiple logistic regression models. In order to assess the predictive hierarchy between different dimensions of variables, associations were consequently examined between the clinical characteristics evaluated by nurses and the admission status after controlling for demographic and emergency service utilization variables (model I), between the clinical characteristics assessed by psychiatrists and the admission status after controlling for demographic and emergency service utilization variables (model II). Full model consisting of demographic, use of emergency service, and clinical characteristics were examined in model III. Hosmer-Lemeshow goodness-of-fit test was used to assess whether these models fitted the data. All statistical analyses were performed using SPSS version 16 (SPSS Inc., Chicago, IL, USA).

## Results

### Demographic and emergency service utilization characteristics

There were 2,777 patients who were admitted after being evaluated in the PES during the study period. Among them, 110 (4%) were coercively hospitalized. Table 1 showed the characteristics of demographics and emergency service utilization in patients of total admissions and those of involuntary admissions. The mean age of the total admitted patients was 42.2±14.6 years. There were no statistically significant differences in the involuntary admission rate in terms of different age strata and gender. Regarding the emergency service utilization pattern, the patients who were compulsively referred by the police had a higher involuntary admission rate. The involuntarily admitted patients tended to be hospitalized during the night shift and stay longer in the PES.

**Table 1:** The characteristics of demographic and emergency service utilization among total admissions and involuntary admissions in the psychiatric emergency service.

Variables	Total admissions	Involuntary admissions		
	N = 2777	n = 110		
	n (%)	n	Rate (%)	*P* value^a^
**Demographic characteristics**				
Gender				0.644
Male	1379 (49.7)	57	4.1	
Female	1398 (50.3)	53	3.8	
Age (year, M±SD)	42.2±14.6	41.9±12.3		0.864
Age				0.225
≦29	554 (20.4)	17	3.1	
30–49	1384 (50.9)	63	4.6	
≧50	783 (28.8)	27	3.4	
**Emergency service utilization pattern**				
Catchment area referral				0.541
Yes	1818 (65.5)	75	4.1	
No	959 (34.5)	35	3.6	
Police referral				<0.001
Yes	1433 (51.6)	84	5.9	
No	1344 (48.4)	26	1.9	
Nighttime arrival				0.355
Yes	1483 (53.4)	54	3.6	
No	1294 (46.6)	56	4.3	
Nighttime discharge				0.075
Yes	76 (2.7)	6	7.9	
No	2701 (97.3)	104	3.9	
Length of stay (hour, M±SD)	20.8±15.4	27.6±14.7		<0.001

^a^ For categorical variables, chi-square test was used to compare the rates of involuntary admission between different categories of each characteristics. For continuous variables, independent-samples t test was used to test the differences in means between voluntary and involuntary admission.

### Clinical characteristics

The clinical characteristics of patients assessed by psychiatric nurses or psychiatrists in total admissions and involuntary admissions were showed in Table 2. Of all the admitted patients, 21.0% had a triage level of III or IV, defined as a higher degree of triage. The patients with a higher level of triage or violence as their presenting problem evaluated by nurses were more likely to be involuntarily admitted. However, there was a lower involuntary admission rate in the patients with anxiety as their presenting problem. Among all admitted patients, the most common diagnosis made by the psychiatrists was psychotic disorders (40.9%), followed by unspecified psychosis (23.0%), bipolar disorder (14.0%), other mood disorders (12.3%), and other diagnoses (9.8%). There were statistically significant differences in the involuntary admission rate between different diagnoses (highest for unspecified psychosis at 5.9%, and lowest for other mood disorders at 2.3%). The percentage of medical consultations or the PICU arrangement among these admitted patients were less than one tenth.

**Table 2:** The clinical characteristics assessed by nurses and psychiatrists among total admissions and involuntary admissions in the psychiatric emergency service.

Variables	Total admission	Involuntary admission		
	N = 2777	n = 110		
	n (%)	n	Rate (%)	*P* value^a^
**Clinical characteristics assessed by nurses**				
Higher triage				<0.001
Yes	583 (21.0)	40	6.9	
No	2194 (79.0)	70	3.2	
Presenting problem				
Violence				<0.001
Yes	769 (27.7)	56	7.3	
No	2008 (72.3)	54	2.7	
Suicide				0.142
Yes	294 (10.6)	7	2.4	
No	2483 (89.4)	103	4.1	
Disturbance				0.345
Yes	1367 (49.2)	59	4.3	
No	1410 (50.8)	51	3.6	
Substance use				0.565
Yes	121 (4.4)	6	5.0	
No	2656 (95.6)	104	3.9	
Anxiety				0.003
Yes	532 (19.2)	9	1.7	
No	2245 (80.8)	101	4.5	
**Clinical characteristics assessed by psychiatrists**				
Diagnosis				0.039
Psychotic disorders^b^	1135 (40.9)	41	3.6	
Unspecified psychosis	639 (23.0)	38	5.9	
Bipolar disorder	390 (14.0)	15	3.8	
Other mood disorders	341 (12.3)	8	2.3	
Others	272 (9.8)	8	2.9	
Physical consultation				0.829
Yes	243 (8.8)	9	3.7	
No	2534 (92.2)	101	4.0	
PICU^c^ admission				0.119
Yes	139 (5.0)	9	6.5	
No	2638 (95.0)	101	3.8	

^a^ For categorical variables, chi-square test was used to compare the rates of involuntary admission between different categories of each characteristics. For continuous variables, independent-samples t test was used to test the differences in means between voluntary and involuntary admission.

^b^ Psychotic disorders were broadly defined and referred to schizophrenia, schizoaffective disorder, and delusional disorder.

^c^ PICU = Psychiatric Intensive Care Unit

### Models assessing the associated factors of involuntary admission

Models evaluating the associations of demographics, emergency service utilization, and clinical characteristics with involuntary admission were shown in Table 3. Full model as in model III showed that neither age nor gender was related to involuntary admission. The associated factors of involuntary admission were police referral (OR = 2.30; 95% CI = 1.43–3.68), inpatient admission during the night shift (OR = 2.72; 95% CI = 1.11–6.67), longer length of stay in the PES (OR = 1.66; 95% CI = 1.31~2.09), and presenting problem as violence (OR = 2.11; 95% CI = 1.37–3.25).

**Table 3:** Models assessing associated factors of involuntary admission in the psychiatric emergency service.

	Model I	Model II	Model III
	OR (95% CI)	OR(95% CI)	OR(95% CI)
**Demographics**			
Male gender	0.91(0.61–1.37)	1.02(0.68–1.52)	0.92(0.61–1.39)
Age (reference: ≦29)			
30–49	1.53(0.87–2.68)	1.56(0.89–2.76)	1.56(0.88–2.77)
≧50	1.14(0.60–2.14)	1.17(0.62–2.21)	1.18(0.62–2.24)
**Emergency service utilization**			
Police referral	2.37(1.49–3.79)*	2.94(1.86–4.63)*	2.30(1.43–3.68)*
Nighttime arrival	0.82(0.55–1.22)	0.83(0.56–1.23)	0.81(0.54–1.21)
Nighttime discharge	2.61(1.07–6.39)*	2.64(1.08–6.43)*	2.72(1.11–6.67)*
Length of stay	1.66(1.32–2.10)*	1.65(1.31–2.08)*	1.66(1.31–2.09)*
**Clinical Characteristics assessed by nurses**			
Higher triage	1.43(0.93–2.21)		1.39(0.90–2.15)
Presenting problem			
Violence	2.03(1.33–3.11)*		2.11(1.37–3.25)*
Suicide	0.89(0.40–2.00)		0.95(0.42–2.13)
Substance	1.20(0.50–2.86)		1.33(0.54–3.25)
Anxiety	0.61(0.30–1.25)		0.65(0.32–1.33)
**Clinical Characteristics assessed by psychiatrists**			
Diagnosis (reference: other mood disorders)			
Psychotic disorders^a^		1.29(0.59–2.81)	1.31(0.60–2.87)
Unspecified psychosis		2.22(1.01–4.90)*	2.17(0.98–4.82)
Bipolar disorder		1.63(0.68–3.93)	1.83(0.75–4.44)
Others		1.25(0.45–3.45)	1.09(0.39–3.09)
Physical consultation		0.86(0.41–1.77)	0.81(0.39–1.70)
PICU^b^ admission		1.79(0.87–3.69)	1.62(0.78–3.35)
Goodness-of-fit^c^	0.65	0.36	0.77

*p<0.05

^a^ Psychotic disorders were broadly defined and referred to schizophrenia, schizoaffective disorder, and delusional disorder.

^b^ PICU = Psychiatric Intensive Care Unit

^c^ *p* value of Hosmer-Lemeshow test

When a comparison was made across three multiple logistic regression models, it could be seen that clinical characteristics assessed by psychiatric nurses were more likely to be associated with involuntary admission than the ones evaluated by psychiatrists. In model II in which only psychiatrists’ evaluations were included in the variables of clinical assessment, patients diagnosed as unspecified psychosis were more likely to be involuntarily placed. However, when evaluations made by nurses were selected into model III, there was no association between psychiatric diagnoses and involuntary admission status. The patients with violence as their presenting problems assessed by nurses were 2.11 times more likely to be related to involuntary admission as shown in Model III, while the demographic and emergency service utilization variables remained somewhat the same significant association level with involuntary admission status across all the regression models. These models all fitted the data as the Hosmer-Lemeshow goodness-of-fit test showed non-significant results (*p* value = 0.65, 0.36, 0.77 for Model I, II, III).

## Discussions

### Principle findings

From January 2009 to December 2010, involuntary admissions accounted for 4% of the admissions in the PES of a psychiatric hospital in Taiwan. The associated factors for involuntary admissions were police referral, violence as the presenting problem assessed by nurses, longer stay in the PES, and psychiatric admission during the night shift.

### The lower proportion of involuntary admission

In this study, the proportion of involuntary admission among those admitted in the PES was 4.0%. This was in the lower range when compared to previous studies, which ranged between 3.2% and 26.4% [1, 7, 8]. This low figure may be due to differences in the assessment criteria and regulations of involuntary admission, Asian culture regarding family involvement in patient admissions in Taiwan, and characteristics of this study cohort.

Heterogeneous assessment criteria of involuntary admission in different countries are applied according to the psychiatric state, dangerous behavior, and need for treatment. In general, psychiatric state instead of psychiatric diagnosis was the main assessment criteria of involuntary admission [7, 8, 10]. In Taiwan, stricter detained criteria such as psychotic state, non-compliance with treatment, and dangerous behavior are the legally mandatory requirements for involuntary admission [11]. However, in the EU, broader detained criteria are applied in the commitment treatment, which include mental illness and dangerous behavior or the need for treatment-criterion [7]. So the lower proportion of involuntary admission in this study could be arisen from the narrower assessment criteria of detainment. In addition, five days are allowed between the emergency placement and approval of the involuntary hospitalization in Taiwan, which is longer than in other countries [7]. During this period, assessments and interventions can be performed by multidisciplinary professionals to enhance patients’ insight into their risky situations, to involve patients and their family members in a collaborative discussion about further treatment, to manage the psychiatric emergency crises, and to arrange appropriate dispositions instead of inpatient treatments. As a result, it is more likely for patients to agree with the admissions during the stay in the PES. As the study site is one of the teaching psychiatric hospitals which is fully staffed with multidisciplinary professionals, lower percentage of involuntary admission could also be attributable to closer adherence to the stricter criteria and complicated procedures of involuntary admission.

Furthermore, Patients’ decisions to inpatient treatment may be greatly influenced by the family members of psychiatric patients in Asia. In Taiwan, the psychiatrists would expect the involvement of family members in the decisions of inpatient treatment. These patients are therefore more likely to sign consent forms of admissions because of the persuasions of their relatives even if they initially refuse to be admitted. In some EU countries, no inclusion of consents from relatives and obligatory inclusion of a legal representative during the commitment procedure are required [7]. This could lead to a lower percentage of involuntary admission in this study. However, in South Korea culturally mandated family referrals can override the patients’ wishes to initiate compulsory admissions, which may account for its high percentage of involuntary admission [9].

Past studies showed that higher proportion of involuntary admission occurred in samples with younger age and male gender [3, 15, 21]. In this study cohort, samples were characterized as older age (42.2 years) and equal gender distribution as compared to previous studies, which could explain the lower percentage of involuntary admissions. However, the proportion of involuntary admission would be raised due to the higher percentage of psychotic disorder or unspecified psychosis (63.9%) and police referrals (51.6%) among this study samples than ones in previous studies [22–24]. In addition, although the ethnicity was not included in the study variables, from clinical experience in Taiwan, the ethnicity characteristics in this study cohort was likely to be highly homogenous as Chinese. This could lead to the lower percentages of involuntary admission than in other cohorts with higher proportions of ethnic minority [16, 22, 25].

### The associated factors of involuntary admission

The most associated factor of involuntary hospitalization was whether the patients were involuntarily referred by the police to the PES. The police officers are the frontline staffs in the community to deal with the imminent danger caused by patients who are uncooperative and refuse to be admitted [21, 23, 26]. Although in Taiwan the police officer have the right to compulsively detain dangerous patients for up to 24 hours under the Police Power Exercise Act [20], initial assessment, emergency placement, short-term detention of dangerous patients in the hospital, and application for involuntary admission are solely performed by the approved psychiatrists as regulated in the Mental Health Act [11]. This regulation is different from the ones in EU countries where other professionals such as the police officers, physicians, social workers, and psychiatric nurses also play their roles in the process of involuntary admission [7]. Our result suggested that the police need to be involved in the evaluation and management of compulsive admission.

Furthermore, the patients with violent behaviors towards others as their presenting problems assessed by psychiatric nurses were associated with involuntary admission. It showed across regression models that this factor was more related to involuntary admission than the clinical diagnoses made by the psychiatrists. Past studies showed that clinical characteristics related to involuntary admission included more severe psychopathology, especially aggressive behavior [16–18]. In psychiatric emergency settings, the psychiatrists would seriously take nurses’ evaluations into consideration when setting treatment plans. Therefore, immediate assessments of the dangerousness from the nurses, especially aggressive behavior, would initiate the psychiatrists to apply for involuntary admission. In the UK, the nurses have the holding power to detain psychiatric patients who are ‘receiving treatment for a mental disorder as in-patients’ for up to six hours [27].

However, the clinical diagnoses made by the psychiatrists were not as important as the evaluations made by the nurses or the referral decisions made by the police. We can see from the regression models that the clinical characteristics evaluated by the psychiatrists were not associated with the involuntary admission status when adjustment of clinical variables assessed by the nurses was made. This finding was inconsistent with previous ones that the patients diagnosed with psychotic disorders [17, 19, 23, 28], especially schizophrenia [15, 29, 30], were more likely to be involuntarily hospitalized. Although in Taiwan, psychosis is one of the legally defined set of required conditions for involuntary admission [11], in the psychiatric emergency room settings, information about the long-term course of psychotic disorder is often hard to be collected. Therefore, tentative diagnoses with low validity such as unspecified psychosis are often made. This could result in the non-association between the psychiatric diagnosis and involuntary admission status in this study. Previous findings regarding other associated diagnoses of involuntary admission, such as dementia, other organic mental disorders, and substance abuse could not be replicated in this study [2, 5, 18].

The involuntarily hospitalized patients tended to stay longer in the PES before they were admitted to inpatient units. As the procedure for compulsive hospitalization is rather complicated and involves patients and their family members, as well as various professionals including the psychiatric nurses, social workers and psychiatrists [8], the duration of stay in the PES tends to be longer before the admissions are arranged. In TCPC, evaluations by two psychiatrists, written opinions from patients assisted by psychiatric nurses, and written opinions from main caregivers assisted by social workers are the clinical pathways leading to involuntary admission. This could be why we found out that more involuntary patients were admitted during the night shift when lengthy applications of involuntary admission had been completed.

There were no statistically significant differences in the rates of involuntary admission with respect to age, gender and presenting problems such as suicide. There were inconsistent results about whether the patients with younger age were more likely to be involuntarily admitted [2, 3]. Male gender has been reported to be associated with involuntary hospitalization in study samples with younger patients [3, 15]. As this study cohort constituted older patients, gender was not related to involuntary admission. In addition, in terms of dangerous behavior, posing danger to others rather than to oneself was more frequently reported to be related with involuntary hospitalization [2, 17, 18]. Patients who manifest with suicidal ideations or behaviors but not with psychotic symptoms are not likely to be involuntarily admitted as the psychotic state is one of the mandatory criteria of involuntary admission in the Mental Health Act of Taiwan [11]. There is also a culturally difference that in Asian countries the role of mental illness in suicide is not as important as that in Western countries [31, 32]. These patients with suicide risk are often managed in the PES and discharged with arrangements of intensive family care.

### Strengths and limitations

The major strength of this study is that it is one of the few studies carried out in non-Western countries [9], with a large sample size to investigate the proportion and related factors of involuntary admission in a PES. Therefore, it can be used as an international reference, especially for countries that are in the process of re-evaluating their commitment criteria and mental health legislation. However, some limitations should also be noted. First, these data were based on real world situations in the PES where the assessment instruments were less validated but based on clinical training and standard of care. Second, since this study used a registered database to analyze, there was a lack of information about some socio-demographic and clinical characteristics related to involuntary admission, including ethnicity [22, 25, 33], socioeconomic status [29], social or family support [24, 29, 34], and social functioning [35]. Other psychiatric service variables, such as adherence to treatment [36, 37], satisfaction with the mental health care they have received [34, 38], frequency of outpatient visits [26], and numbers of previous involuntary hospitalizations [12, 26, 36, 38], were also not included in this study. A broader issue on ecological factors related to compulsive admission, such as integrated psychiatric services [39], community-care networks [40], and country’s economy [13] may also be taken into consideration. Other statistics such as outpatient, hospital episode, and national information are suggested to be included in future studies to overcome the limited coverage of the PES database. Third, although this study included 43.5% of all involuntary admissions in Taipei City and was representative of the urban PES in the developed countries, the findings cannot be directly generalized to other regions or countries with different backgrounds such as rural areas, countries with different Mental Health Act, and developing countries.

## Conclusions

This study showed a lower involuntary admission proportion in a psychiatric emergency service in Taiwan. Multidisciplinary collaboration is suggested as an important element in the process of compulsive admission, during which both the police and nurses can offer initial assessment to assist the decisions on involuntary admissions. Therefore, it is critical to provide training for multidisciplinary professionals in hospital and community settings about how and what to evaluate for better results during involuntary admission. Due to a greater deal of time and efforts being spent before the involuntary admission, more manpower and resources are suggested to be allocated in the psychiatric emergency services where comprehensive evaluations and interventions can be done.

## References

[1] Salize HJ, Dressing H (2004) Epidemiology of involuntary placement of mentally ill people across the European Union. Br J Psychiatry. 184: 163–168. 14754830 10.1192/bjp.184.2.163

[2] Mulder CL, Uitenbroek D, Broer J, Lendemeijer B, van Veldhuizen JR, van Tilburg W, et al (2008) Changing patterns in emergency involuntary admissions in the Netherlands in the period 2000–2004. Int J Law Psychiatry. 31: 331–336. 10.1016/j.ijlp.2008.06.007 18667238

[3] Lelliott P, Audini B (2003) Trends in the use of Part II of the Mental Health Act 1983 in seven English local authority areas. Br J Psychiatry. 182: 68–70. 12509321 10.1192/bjp.182.1.68

[4] Hotopf M, Wall S, Buchanan A, Sessely S, Churchill R (2000) Changing pattern in the use of the Mental Health Act 1983 in England, 1984–1996. Br J Psychiatry. 176: 479–484. 10912226 10.1192/bjp.176.5.479

[5] Keown P, Mercer G, Scott J (2008) Retrospective analysis of hospital episode statistics, involuntary admissions under the Mental Health Act 1983, and number of psychiatric beds in England 1996–2006. BMJ. 337: a1837 10.1136/bmj.a1837 18845592 PMC2565753

[6] Keown P, Weich S, Bhui KS, Scott J (2011) Association between provision of mental illness beds and rate of involuntary admissions in the NHS in England 1988–2008: ecological study. BMJ. 343: d3736 10.1136/bmj.d3736 21729994 PMC3130113

[7] Dressing H, Salize HJ (2004) Compulsory admission of mentally ill patients in European Union Member States. Soc Psychiatry Psychiatr Epidemiol. 39: 797–803. 15669660 10.1007/s00127-004-0814-9

[8] Zinkler M, Priebe S (2002) Detention of the mentally ill in Europe-a review. Acta Psychiatr Scand. 106: 3–8. 12100342 10.1034/j.1600-0447.2002.02268.x

[9] Bola JR, Park EH, Kim SY (2011) Reassessing the high proportion of involuntary psychiatric hospital admissions in South Korea. Community Ment Health J. 47: 603–606. 10.1007/s10597-011-9396-7 21416122

[10] Riecher-Rössler A, Rössler W (1993) Compulsory admission of psychiatric patients—an international comparison. Acta Psychiatr Scand. 87: 231–236. 8488742 10.1111/j.1600-0447.1993.tb03363.x

[11] Department of Health, Taiwan (2007) Mental Health Act. Available: http://law.moj.gov.tw/Eng/LawClass/LawContent.aspx?PCODE=L0020030/ Accessed 2014 Sep 29.

[12] Fennig S, Rabinowitz J, Fennig S (1999) Involuntary first admission of patients with schizophrenia as a predictor of future admissions. Psychiatr Serv. 50(8): 1049–1052. 10445653 10.1176/ps.50.8.1049

[13] Kessell ER, Catalano RA, Christy A, Monahan J (2006) Rates of unemployment and incidence of police-initiated examinations for involuntary hospitalization in Florida. Psychiatr Serv. 57(10): 1435–1439. 17035561 10.1176/ps.2006.57.10.1435

[14] Beveridge R, Clarke B, Janes L, Savage N, Thompson J, Dodd G, et al (1999) Canadian emergency department triage and acuity scale implementation guidelines. CJEM 1(3 Suppl): s1–24.

[15] Cougnard A, Kalmi E, Desage A, Misdrahi D, Abalan F, Brun-Rousseau H, et al (2004) Factors influencing compulsory admission in first-admitted subjects with psychosis. Soc Psychiatry and Psychiatr Epidemiol. 39: 804–809. 15669661 10.1007/s00127-004-0826-5

[16] Mulder CL, Koopmans GT, Selten JP (2006) Emergency psychiatry, compulsive admissions and clinical presentation among immigrants to the Netherlands. Br J Psychiatry. 188: 386–391. 16582067 10.1192/bjp.188.4.386

[17] Singh SP, Croudace T, Beck A, Harrison G (1998) Perceived ethnicity and the risk of compulsory admission. Soc Psychiatry and Psychiatr Epidemiol. 33: 39–44. 9448444 10.1007/s001270050020

[18] Schuepbach D, Goetz I, Boeker H, Hell D (2006) Voluntary vs. involuntary hospital admission in acute mania of bipolar disorder: Results from the Swiss sample of the EMBLEM study. J Affect Disord. 90: 57–61. 16324749 10.1016/j.jad.2005.09.012

[19] Myklebust LH, Sørgaard K, Røtvold K, Wynn R (2011) Factors of importance to involuntary admission. Nord J Psychiatry. 66: 178–182. 10.3109/08039488.2011.611252 21936731

[20] Ministry of the Interior, Taiwan (2011). Police Power Exercise Act. Available: http://law.moj.gov.tw/Eng/LawClass/LawContent.aspx?PCODE=D0080145/ Accessed 2014 Sep 29.

[21] Douzenis A, Michopoulos I, Economou M, Rizos E, Christodoulou C, Lykouras L (2010) Involuntary admission in Greece: A prospective national study of police involvement and client characteristics affecting emergency assessment. Int J Soc Psychiatry. 58(2): 172–177. 10.1177/0020764010387477 21106603

[22] Morgan C, Mallett R, Hutchinson G, Bagalkote H, Morgan K, Fearon P, et al (2005) Pathways to care and ethnicity. 1: Sample characteristics and compulsory admission. Report from the AESOP study. Br J Psychiatry. 186: 281–289. 15802683 10.1192/bjp.186.4.281

[23] van der Post L, Visch I, Mulder C, Schoevers R, Dekker J, Beekman A (2012) Factors associated with higher risks of emergency compulsory admission for immigrants: a report from the ASAP study. Int J Soc Psychiatry. 58(4): 374–380. 10.1177/0020764011399970 21628357

[24] van der Post LF, Mulder CL, Peen J, Visch I, Dekker J, Beekman AT (2012) Social support and risk of compulsive admission: part IV of the Amsterdam study of acute psychiatry. Psychiatr Serv. 63(6): 577–583. 10.1176/appi.ps.201100080 22638005

[25] Davies S, Thornicroft G, Leese M, Higgingbotham A, Phelan M (1996) Ethnic differences in risk of compulsory psychiatric admission among representative cases of psychosis in London. BMJ. 312: 533–537. 8595280 10.1136/bmj.312.7030.533 PMC2350333

[26] van der Post L, Mulder CL, Bernardt CML, Schoevers RA, Beekman ATF, Dekker J (2009) Involuntary admission of emergency psychiatric patients: report from the Amsterdam study of acute psychiatry. Psychiatr Serv. 60(11): 1543–1546. 10.1176/appi.ps.60.11.1543 19880477

[27] Department of Health, Great Britain (2008) Code of Practice: Mental Health Act 1983.

[28] Hansson L, Muus S, Saarento O, Vinding HR, Göstas G, Sandlund M, et al (1999) The Nordic comparative study on sectorized psychiatry: rates of compulsory care and use of compulsory admissions during a 1-year follow-up. Soc Psychiatry Psychiatr Epidemiol. 34: 99–104. 10189816 10.1007/s001270050118

[29] Riecher A, Rössler W, Löffler W, Fätkenheuer B (1991) Factors influencing compulsory admission of psychiatric patients. Psychol Med. 21: 197–208. 2047496 10.1017/s0033291700014781

[30] Bebbington PE, Feeney ST, Flannigan CB, Glover GR, Lewis SW, Wing JK (1994) Inner London collaborative audit of admissions in two health districts. II: Ethnicity and the use of the Mental Health Act. Br J Psychiatry. 165: 743–749. 7881776 10.1192/bjp.165.6.743

[31] Chen YY, Wu KCC, Yousuf S, Yip PSF (2012) Suicide in Asia: opportunities and challenges. Epidemiol Rev. 34: 129–144. 10.1093/epirev/mxr025 22158651

[32] Chen YY, Yip PSF (2008) Rethinking suicide prevention in Asian countries. Lancet. 372: 1629–1630. 10.1016/S0140-6736(08)61678-5 18994653

[33] Thomas CS, Stone K, Osborn M, Thomas PF, Fisher M (1993) Psychiatric morbidity and compulsory admission among UK-born Europeans, Afro-Caribbeans and Asians in central Manchester. Br J Psychiatry. 163: 91–99. 8353706 10.1192/bjp.163.1.91

[34] van der Post LF, Peen J, Dekker JJ (2014) A prediction model for the incidence of civil detention for crisis patients with psychiatric illnesses: the Amsterdam study of acute psychiatry VII. Soc Psychiatry Psychiatr Epidemiol. 49: 283–290. 10.1007/s00127-013-0742-7 23863912

[35] Lindsey MA, Joe S, Muroff J, Ford BE (2010) Social and clinical factors associated with psychiatric emergency service use and civil commitment among African-American youth. Gen Hosp Psychiatry 32: 300–309. 10.1016/j.genhosppsych.2010.01.007 20430234 PMC2862230

[36] Oluwatayo O, Gater R (2004) The role of engagement with services in compulsory admission of African/Caribbean patients. Soc Psychiatry Psychiatr Epidemiol. 39: 739–743. 15672295 10.1007/s00127-004-0794-9

[37] Schuepbach D, Novick D, Haro JM, Reed C, Boeker H, Noda S, et al (2008) Determinants of voluntary vs. involuntary admission in bipolar disorder and the impact of adherence. Pharmacopsychiatry. 41: 29–36. 10.1055/s-2007-993213 18203049

[38] van der Post LF, Peen J, Visch I, Mulder CL, Beekman AT, Dekker JJ (2014). Patient perspectives and the risk of compulsive admission: the Amsterdam study of acute psychiatry V. Int J Soc Psychiatry. 60(2): 125–133. 10.1177/0020764012470234 23333906

[39] Wierdsma AI, Mulder CL (2009) Does mental health service integration affect compulsory admissions? Int J Integr Care. 9: e90 19777114 10.5334/ijic.324 PMC2748183

[40] Wierdsma AI, Poodt HD, Mulder CL (2007) Effects of community-care networks on psychiatric emergency contacts, hospitalization and involuntary admission. J Epidemiol Community Health. 61: 613–618. 17568054 10.1136/jech.2005.044974 PMC2465738

